# Ethics of artificial intelligence-assisted image interpretation in dermatopathology

**DOI:** 10.1016/j.jdin.2025.01.005

**Published:** 2025-01-27

**Authors:** Hayden Smith, Travis Blalock, Benjamin K. Stoff

**Affiliations:** a Department of Dermatology, Emory University School of Medicine, Atlanta, Georgia; b Emory Center for Ethics, Emory University School of Medicine, Atlanta, Georgia

**Keywords:** artificial intelligence, beneficence, ChatGPT-4, dermatopathology, ethics, health literacy, justice, pathology report, trust

*To the Editor:* There is significant interest in artificial intelligence (AI)-assisted image interpretation in medicine. AI tools have increased diagnostic accuracy and efficiency in radiology and anatomic pathology and are showing great promise in clinical dermatology and dermatopathology.^1^ These AI technologies have also shown tremendous accuracy in diagnosing melanoma, nonmelanoma skin cancer, and specific skin conditions.^2^ However, when contemplating the best way to incorporate AI into dermatopathology practice, dermatopathologists should consider the principles of beneficence, justice, nonmaleficence, and trust.

AI can enhance radiological image pattern recognition, report generation, and formulate likelihoods for differential diagnoses. AI improved the likelihood of detecting breast cancers in early stages by highlighting subtle pathologic regions and encouraging radiologists to inspect these regions carefully.^1^ AI can be applied in the same way to dermatopathology images. AI very accurately diagnosed nodular basal cell carcinoma, seborrheic keratosis, and dermal nevus after completing a comprehensive training set.^2^ Additionally, a recent systematic review revealed impressive diagnostic accuracy using deep learning algorithms to diagnose specific skin conditions and melanoma.^2^ Evidence in the literature strongly supports the potential for AI tools to improve diagnostic accuracy in dermatopathology, with a likely improvement in patient care (beneficence).

AI-assisted imaging interpretation also has the potential to improve dermatopathology education. In a group of pathologists and pathology residents, AI yielded improved evaluation of digital pathology images of breast biopsies, maximizing pathologists’ interobserver agreements and diagnostic accuracies.^1^ Similar use of AI in dermatopathology could improve education for trainees by reinforcing a confident diagnosis that benefits patients (beneficence).

AI has also been shown to enhance patient understanding of radiology image reports.^3^ When applied to a dermatopathology report, this tool may increase patients’ understanding of diagnosis, especially among those with limited health literacy or poor access to health care (beneficence, justice).

However, AI image interpretation in dermatopathology raises ethical concerns regarding professional services. AI-based image interpretation designed to detect malignancy based on cellular density sometimes classified benign structures such as glands and hair follicles as likely melanoma.^4^ Overdiagnosis or underdiagnosis negatively impacts patients (maleficence). However, even the gold standard for diagnosis in dermatology, dermatopathologist interpretation, is imperfect.

Some AI technology also requires access to protected health information. Therefore, transparency regarding use of AI for diagnosis and data analysis is ethically imperative (trust). However, some argue that AI is so integrated into numerous applications that disclosing every instance of its use may not be practical.

As in radiology and other specialties, AI can augment diagnosis of dermatopathology specimens. Dermatopathologists would be wise to not rely exclusively on AI programs for diagnosis and to verify AI-generated interpretations. Additionally, when feasible, disclosing the use of AI to patients is important to protect patient autonomy. Informed consent may not be imperative (and is often impractical) in this circumstance.^5^ Future research is needed to confirm the direct effect of AI tools on patient experiences and patient outcomes, the potential for error and misdiagnosis, and the responsible use of protected health information.

## Conflicts of interest

None disclosed.
